# Hospital deaths and adverse events in Brazil

**DOI:** 10.1186/1472-6963-11-223

**Published:** 2011-09-19

**Authors:** Mônica Martins, Claudia Travassos, Walter Mendes, Ana Luiza B Pavão

**Affiliations:** 1 Departamento de Administração e Planejamento em Saúde - Escola Nacional de Saúde Pública Sérgio rouca. Fundação Oswaldo Cruz. Rio de Janeiro, RJ, Brazil; 2 Laboratório de Informações em Saúde - Instituto de Comunicação e Informação Científica e Tecnológica em Saúde. Fundação Oswaldo Cruz. Rio de Janeiro, RJ, Brazil

## Abstract

**Background:**

Adverse events are considered a major international problem related to the performance of health systems. Evaluating the occurrence of adverse events involves, as any other outcome measure, determining the extent to which the observed differences can be attributed to the patient's risk factors or to variations in the treatment process, and this in turn highlights the importance of measuring differences in the severity of the cases. The current study aims to evaluate the association between deaths and adverse events, adjusted according to patient risk factors.

**Methods:**

The study is based on a random sample of 1103 patient charts from hospitalizations in the year 2003 in 3 teaching hospitals in the state of Rio de Janeiro, Brazil. The methodology involved a retrospective review of patient charts in two stages - screening phase and evaluation phase. Logistic regression was used to evaluate the relationship between hospital deaths and adverse events.

**Results:**

The overall mortality rate was 8.5%, while the rate related to the occurrence of an adverse event was 2.9% (32/1103) and that related to preventable adverse events was 2.3% (25/1103). Among the 94 deaths analyzed, 34% were related to cases involving adverse events, and 26.6% of deaths occurred in cases whose adverse events were considered preventable. The models tested showed good discriminatory capacity. The unadjusted odds ratio (OR 11.43) and the odds ratio adjusted for patient risk factors (OR 8.23) between death and preventable adverse event were high.

**Conclusions:**

Despite discussions in the literature regarding the limitations of evaluating preventable adverse events based on peer review, the results presented here emphasize that adverse events are not only prevalent, but are associated with serious harm and even death. These results also highlight the importance of risk adjustment and multivariate models in the study of adverse events.

## Background

The occurrence of adverse events is considered a serious problem worldwide regarding the performance of health services [1]. A report by the United States Institute of Medicine called attention to the fact that the mortality rate due to adverse events was higher than that attributed to some of the principal causes of mortality, such as motor vehicle accidents, breast cancer, and AIDS [2]. Studies have indicated that approximately 10% of the incidence of adverse events are related to hospital care, and it is estimated that 4.4% to 20.8% of adverse events lead to patient death [3,4].

The concept of adverse event is related to the occurrence of harm or injury caused by medical care rather than by the underlying disease [5]. Thus, assessing the occurrence of an adverse event involves distinguishing between undesirable results caused by problems in the quality of care and those resulting from the patient's inherent risk factors and the severity of the case, which define the prognosis and odds of survival [6,7]. A central concern in evaluating the outcomes of health care, especially regarding adverse events or hospital deaths, is the identification of preventable cases [8].

Sox et al [9] contrast the Institute of Medicine's estimate of deaths of hospitalized patients resulting from medical error [2] with the robustness of the methods used in this calculation. Their central argument is that the methodology involves peer review, characterized by a high degree of subjectivity in the assessment and low inter-reviewer reliability [8].

Few studies were carried out with the particular purpose of identifying an association between hospital deaths and adverse events [3,4,10,11]. Generally, studies have focused on evaluating specific situations such as surgical cases and hospital infections [10,12,13]. Garcia-Martin et al [11] developed a case-control study to estimate the proportion of hospital deaths associated with adverse events. They found that the presence of at least one adverse event was significantly associated with an increased risk of death (OR = 1.75), and that 24% of deaths were associated with adverse events. A study carried out in the Netherlands [3] examined adverse events and preventable deaths using a specific sample of cases of death and found that, among the patients who died, 10.7% had suffered an adverse event, while preventable adverse events accounted for 4.1% of hospital deaths. Importantly, Hayward & Hofer [8] point to an overestimation of preventable deaths associated with good-quality care, since the majority of estimates overlooks the underlying prognostic factors of patients who died. Weingart et al [14] further highlight that risk is not homogeneous. Patients who are more severely ill, are subject to multiple interventions and remain hospitalized longer are more likely to suffer serious injury or harm as a result of medical error.

Considering the relevance of this theme and the lack of studies, especially in developing countries, this article has the objective to evaluate the relationship between hospital deaths and adverse events, adjusted for patient risk factors, in hospitalized patients in Brazil.

## Methods

### Study design and population

The data were obtained from a previous study on the incidence of adverse events in Brazilian hospitals that used retrospective chart review as the methodology [15,16], based on the design and tools developed by Canadian researchers. According to the Canadian Adverse Event Study [17], an adverse event was defined as an unintended injury which results in temporary or permanent disability, including increased length of stay caused by clinical care rather than disease process. Preventability refers to an adverse event resulting from an error or series of errors, either negligent or non-negligent, or failure.

Patient charts were reviewed in two stages: (1) screening of potential adverse events by nurses and (2) identification of adverse events by physicians. The screening stage was based on the presence of at least one of 18 previously established screening criteria (trigger tools). Death was one of the 18 triggers, so all the deaths were selected for the second stage. Based on evidence in each patient's chart, the second stage of evaluation confirmed (or disproved) the occurrence of an adverse event and its preventability. Six-point scales were used, and results greater than or equal to 4 were defined as confirming the presence of an adverse event and its preventability. The assessment of preventable adverse events was based on subjective and implicit criteria, backed by the expertise, practical experience and clinical decision-making of physicians [18]. The assumption was that a preventable adverse event results from poor quality in the process of care or problems in the health system.

In the present study, 1103 patient charts were selected by means of simple random sampling out of 27,350 adult patients admitted between January 1^st^, 2003, and December 31^st^, 2003, to 3 general teaching hospitals in the state of Rio de Janeiro, Brazil. Two of the hospitals have emergency departments and maternity wards. The hospitals were selected based on their voluntary willingness to collaborate and the degree of excellence in their areas of expertise.

Following the Canadian study [17], the exclusion criteria were patients under 18 years of age, patients who remained hospitalized for less than 24 hours, and psychiatric patients. However, unlike the Canadian study, obstetric cases were included in the sample in face of the persistently high maternal mortality rate in Brazil. The random sample process was applied to each hospital. The parameters used to define the sample size were also based on the Canadian study: a 10% expected incidence of patients with adverse events (maximum absolute error -3%), a 50% proportion of potential adverse events, and a significance level of 5%. The loss rate was estimated at 10%, and the rate of ineligible patients was estimated at 20% [16].

### Data analysis

The analysis of the relationship between deaths and adverse events assumed that patient characteristics associated with case severity are important confounding factors. In order to identify the variables associated with the risk of death in the study population, we conducted univariate and bivariate analyses using variables related to demographic and clinical patient characteristics and to the hospitalization, including type of admission (elective, urgency, and emergency), specialty (obstetrics versus non-obstetrics), procedure performed, and length of stay. Patients' social variables were not analyzed due to the high percentage of missing information.

Multiple logistic regression was used to analyze factors associated with death in the selected cases. The dichotomous dependent variable was death (yes/no). Only deaths that occurred during the hospital stay were analyzed. The following steps were applied for modeling: (1) baseline model; (2) introduction of the variables related to the process of care: clinical specialty (obstetrics: yes/no), length of stay (continuous variable), and performance of a surgical or invasive procedure (yes/no); (3) introduction of the occurrence of an adverse event.

The initial baseline model (model 1) was constructed to adjust for patient risk factors, since the prognosis depends on patient attributes that indicate the severity of the case. The baseline model consisted of the following independent variables: age, gender, primary diagnosis, secondary diagnoses (comorbidities), and type of admission. Age was treated as a categorical variable, where younger than 50 years of age was the reference category, and patients older than 79 were grouped due to the small number of cases. Gender, treated as a dichotomous variable, used the male gender as the reference category. This variable was maintained in the model for theoretical reasons, although it has not shown to be of statistical significance.

The primary diagnosis of patients is an essential dimension for risk adjustment, but specific reasons for admission were not considered due to the wide range of diagnostic categories identified and the low number of cases in each category. As a strategy to control severity, the Charlson index [19] was applied to the primary diagnosis. The variable with the Charlson index score for the primary diagnosis was treated as a dichotomous variable, where the reference category was a score equal to 0.

The Charlson index [19] was also applied to the 6 reported secondary diagnoses and was the fourth independent variable introduced into the baseline model. This index was treated as a categorical variable. According to the frequency distribution, the categories for this index were regrouped as follows: (1) score 0 (reference category); (2) score 1; and (3) score greater than or equal to 2. We used the algorithm developed by Quan et al [20] to translate the clinical conditions from the Charlson index [19] to the International Classification of Diseases, 10^th ^Revision (ICD-10).

Type of admission was the last variable included in the baseline model, since unplanned admissions (emergencies) generally entail greater risk of death than planned (elective) admissions [7]. Type of admission was treated as a dichotomous variable with 2 groups, where elective admissions constituted the reference category.

Variables related to the process of care - length of stay, surgical or invasive procedure performed, and specialty - were also tested (model 2). Length of stay was treated as a continuous variable. The reference categories for procedures performed and specialty were no procedure performed and obstetrics, respectively. These variables were subsequently excluded because they lacked statistical significance.

The occurrence of an adverse event, including its preventability, was the main independent variable for measuring whether this event represented a risk factor associated with the worsening of patient prognosis and an adverse outcome (death). It was included in the third modeling step (final model). The occurrence of adverse events was treated as a categorical variable, and the categories were grouped as follows: (1) no adverse events (reference category); (2) no preventable adverse events; and (3) preventable adverse events. The cases of adverse events are described in the Additional File 1: Appendix.

We tested the impact of the introduction of each variable on the model's performance, starting from the baseline model. The adequacy of the model for predicting death was assessed according to the percentage of improvement in the model as compared to the initial deviance (likelihood χ^2^), C statistics, and Hosmer-Lemeshow test. The statistical analyses were processed with SPSS 17.0. This study was approved by the Ethics Committee of the Oswaldo Cruz Foundation, protocol 271/05.

## Results

A total of 1103 clinical charts of patients admitted to 3 teaching hospitals in the state of Rio de Janeiro were reviewed. Most admissions were in non-obstetric specialties (80.5%). The mean age was 46.9 years, and 61.3% of the patients admitted were women (Table 1). The proportions of principal diagnoses grouped according to the chapters of the ICD-10 revealed that 44.7% of the cases analyzed were admitted for problems associated with pregnancy, childbirth and postpartum, diseases of the circulatory system, and diseases of the digestive system. The proportion of admissions with a secondary diagnosis recorded in the charts was 54.2%, while in cases of death this proportion was 87.2% (Table 1). More than 50% of the cases were emergency admissions; more than 70% of the cases underwent some procedure. Of the 94 deaths analyzed, 34% occurred in cases with an adverse event, and 26.6% of the deaths occurred in cases involving adverse events classified as preventable (Table 1).

**Table 1:** Study population characteristics: sampled admissions and deaths

Characteristics	Sampled cases	Deaths
**Number of cases **(% of total)	1103 (100.0)	94 (8.5)
**Demographics**		
Age (years)		
Mean age in years (SD)	46.9 (19.1)	61.9 (16.7)
Median	46	65
Mode (Range)	25 (18-92)	71 (20-92)
Age bracket (%)		
18-49	56.4	21.3
50-59	14.0	18.1
60-69	13.9	23.4
70-79	11.1	23.4
80-99	4.6	13.8
Women (%)	61.3	47.9
**Comorbidity**		
Record of 1 secondary diagnosis (%)	54.2	87.2
Record of 2 secondary diagnoses (%)	33.0	75.5
Record of 6 secondary diagnoses (%)	2.5	8.5
**Charlson index **(% by score)		
Primary diagnosis		
0	82.0	57.4
1	18.0	42.6
Secondary diagnoses		
0	75.1	46.8
1	13.2	24.5
≥ 2	11.7	28.7
**Type of admission **(%)		
Elective	44.6	14.9
Urgency/Emergency	55.4	85.1
**Specialty **(%)		
Obstetrics	19.5	0
Non-obstetrics	80.5	100.0
**Procedure performed **(%)	77.5	72.3
**Length of stay **(days)		
Mean (SD)	12.8 (23.6)	25.2 (33.4)
Median	5.0	16.5
Mode	2.0	7.0*
Range	1-408	1-236
**Adverse event**		
Number of patients (% of total)	84 (7.6)	32 (34.0)**
**Preventable adverse event**		
Number of patients (% of total)	56 (5.1)	25 (26.6) **
**Non-preventable adverse event**		
Number of patients (% of total)	28 (2.5)	7 (7.5) **

* There is more than one value considered as the mode.

** Proportional mortality.

Analysis of the relationship between death and patient risk factors (age, gender, Charlson index) showed a statistically significant association (Table 2). Except for gender, the correlations were around 0.20. Type of admission had a correlation of 0.21 and unadjusted odds ratio of 5.14 (Table 2). Among the variables related to the process of care (specialty, procedure, and length of stay), only the type of procedure was not statistically significant.

**Table 2:** Measures of association between death and patient characteristics, process of care, preventable and non-preventable adverse events

Independent variables	Number of cases (A)	Number of deaths (B)	% Deaths (B/A*100)	Measure of association
**Gender**				
Male*	427	49	11.5	OR 0.55 (95% CI: 0.36-0.84)
Female	676	45	6.7	(p < 0.005) R = -0.08
**Age bracket**				
18-49*	622	20	3.2	
50-59	155	17	11.0	χ2 63.380; 4 df, p < 0.000
60-69	153	22	14.4	Somers' D 0.11, p < 0.000
70-79	122	22	18.0	R = 0.24
80-99	51	13	25.5	
**Charlson index (score)**				
Primary diagnosis				
0*	904	54	6.0	OR 3.96 (95% CI: 2.54-6.16)
1	199	40	20.1	(p < 0.000) R = 0.20
Secondary diagnosis				
0*	828	44	5.3	χ2 46.200; 2 df, p < 0.000
1	146	23	15.8	Somers' D 0,12, p < 0.000
≥ 2	129	27	20.9	R = 0.20
**Type of admission**				
Elective*	492	14	2.8	OR 5.14 (95% CI: 02.89-9.20)
Urgency/Emergency	611	80	13.1	(p < 0.000) R = 0.18
**Specialty**				
Obstetrics*	215	0	0.0	OR 0.79 (95% CI: 0.76-0.81)
Non-obstetrics	888	94	10.6	p < 0.000 R = 0.15
Procedures performed**				
No*	248	26	10.5	OR 0.74 (95% CI: 0.46-1.19)
Yes	855	68	8.0	(p > 0.209) R = -0.04
Adverse event*				
No *	1019	62	6.1	OR 9.50 (95% CI: 5.71-15.82) (p < 0.000)
Yes	84	32	38.1	R = 0.30
**Preventable adverse event**				
No*	1047	69	6.6	OR 11.43 (95% CI: 6.40-20.43) (p < 0.000)
Yes	56	25	44.6	R = 0.30

**Total**	**1103**	**94**	**8.5**	

Dependent variable: death.

OR: Odds ratio; R: Pearson correlation.

* Reference categories for odds ratios.

** Included surgical and invasive procedure.

The overall hospital mortality rate was 8.5%, but this rate increased to 38.1% in the subset of cases with the occurrence of an adverse event, and to 44.6% in the subset of cases with a preventable adverse event (Table 2). The correlation between death and adverse event showed a statistically significant association, with odds ratio of 9.50 (Table 2). There was also a significant association between death and preventable adverse event, with an even higher odds ratio (11.43). The mortality rate related to adverse events was 2.9% (32/1103), while that related to preventable adverse events was 2.3% (25/1103).

The mean length of stay, which may reflect a consequence of the adverse event and the severity of the case, was 25.1 days in cases with a fatal outcome (SD: 33.4; 95% CI: 18.3-32.0 days), more than twice as much as cases without death (11.1 days - SD: 22.2; 95% CI: 9.7-12.5 days). A similar pattern emerged when comparing cases with and without an adverse event: the mean length of stay tripled in cases with adverse events (32.4 days - SD: 36.0; 95% CI: 24.6-40.2 days) compared to those without adverse events (10.6 days - SD: 21.5; 95% CI: 9.3-12.0 days).

None of the tested models showed any adjustment problems, as confirmed by the Hosmer-Lemeshow test (Table 3). The three models presented good predictive capacity, as measured by the C statistic (Table 3). The introduction of the variable related to the occurrence of an adverse event impacted the model's performance. Among the variables related to the process of care, length of stay was the only one statistically significant, but it did not impact the baseline model's discriminatory capacity (Table 3; model 2). In the second modeling step, the introduction of the clinical specialty variable (obstetrics, yes/no) was not statistically significant, besides slightly decreasing the model's predictive capacity (C statistic = 0.84; 95% CI 0.80-0.88).

**Table 3:** Discriminatory capacity and adjustment of models for predicting death

Model	χ^2 ^of Model*	Hosmer-Lemeshow χ^2 ^test	C Statistic (95% CI)
1. Baseline model**:	137.236 (df 9, sig. 0.000)	13.898 (sig. 0.053)	0.85 (0.82-0.89)
2. Baseline model + Length of stay***	148.015 (df 10, sig. 0.000)	13.638 (sig. 0.092)	0.85 (0.82-0.89)
3. Final model: Model 2 + Occurrence of adverse event ****	187.316 (df 12, sig. 0.000)	11.271 (sig. 0.187)	0.88 (0.85-0.91)

* Initial likelihood χ^2 ^with intercept: 484.387 (Wald).

** Baseline model includes: gender, age, Charlson index applied to primary and secondary diagnoses, and type of admission.

*** Surgical or invasive procedure and clinical specialty variables were also tested, but were excluded due to lack of statistical significance.

**** Occurrence of adverse event = no, yes non-preventable, yes preventable.

With the exception of gender, the odds ratios for the other variables were greater than 1.5. The application of the Charlson index to the primary diagnosis yielded an odds ratio of 2.41 for cases with score greater than or equal to 1, as compared to score equal to 0. This index also displayed an upward gradient when applied to comorbidities (Table 4, baseline model). Age greater than 79 years (OR: 8.18) and unplanned admissions had the highest odds ratios in the baseline model (Table 4). In the baseline and final models, score 1 in the Charlson index applied to comorbidities was not statistically significant (Table 4). In the final model, risk of death adjusted by case severity was high in patients with non-preventable adverse events (OR 6.23), compared to patients without any adverse event. However, patients with an adverse event classified as preventable showed an even higher odds ratio (8.23) (Table 4). Regarding patient risk variables in the final model, after the occurrence of adverse events was included, the odds ratio for elderly patients (> 79 years) presented the biggest decrease. For the other variables the odds ratio remained quite similar, being slightly lower for patients between 50 and 59 years old (Table 4).

**Table 4:** Logistic regression for prediction of hospital death: baseline model and final model

Baseline model	β	p-value	Odds ratio	95% CI	
Female gender	-0.572	0.018	0.56	0.35	0.91
Age bracket (years)					
18-49*		0.000			
50-59	0.961	0.011	2.61	1.25	5.47
60-69	1.359	0.000	3.89	1.93	7.85
70-79	1.388	0.000	4.01	1.97	8.13
80-99	2.102	0.000	8.18	3.54	18.90
Primary diagnosis (Charlson index ≥ 1)	0.879	0.000	2.41	1.47	3.94
Secondary diagnoses (Charlson index)					
0*		0.009			
1	0.476	0.122	1.61	0.88	2.95
2	0.895	0.002	2.45	1.37	4.36
Urgency and emergency admissions	1.750	0.000	5.76	3.12	10.61
Constant	-4.603	0.000	0.01		
					

**Final Model**	**β**	**p-value**	**Odds ratio**	**95% CI**	

Female gender	-0.662	0.011	0.52	0.31	0.86
Age bracket (years)					
18-49*		0.000			
50-59	0.993	0.010	2.70	1.26	5.77
60-69	1.115	0.003	3.05	1.45	6.40
70-79	1.178	0.002	3.25	1.53	6.88
80-99	1.879	0.000	6.55	2.63	16.32
Primary diagnosis (Charlson index ≥ 1)	0.859	0.001	2.36	1.40	3.98
Secondary diagnoses (Charlson index)					
0*		0.013			
1	0.523	0.107	1.69	0.89	3.18
2	0.893	0.004	2.44	1.33	4.49
Urgency and emergency admissions	1.788	0.000	5.98	3.14	11.39
Length of stay	0.008	0.044	1.01	1.00	1.02
Occurrence of adverse event					
No*					
Yes, non-preventable	1.832	0.001	6.25	2.21	17.70
Yes, preventable	2.108	0.000	8.23	4.02	16.82
Constant	-4.957	0.000	0.01		

* Reference categories

## Discussion

The occurrence of adverse events was examined as a factor associated with risk of death adjusted by case severity. The overall mortality rate in the study population was 8.5%; 2.9% had adverse events and 2.3% had preventable adverse events. The logistic regression models tested in order to evaluate the relationship between hospital death and adverse event, adjusted according to patient risk, showed good discrimination capacity [21]. The unadjusted odds ratio between death and preventable adverse event was high (OR 11.43), while the risk of dying adjusted for patient risk factors decreased to 8.23, but remained high. The relationship between death and non-preventable adverse event, i.e. complications due to the complexity of the case, showed an even high adjusted odds ratio (OR 6.25).

More than ten years ago, Hayward & Hofer [8,22] highlighted the fact that estimates on the relationship between death and adverse event failed to consider patient risk factors in the analysis. In the present study, using logistic regression for risk adjustment, it can be seen that older patients have higher odds of dying. However, it appears that part of this risk is related to the occurrence of adverse events. This point demonstrates the difficulty of isolating the effect of each factor. It seems that this kind of association is much more synergic and involves patient risk factors, the process of care, non-preventable complications, and even preventable adverse events. Nevertheless, 50-59 year-old patients in our study presented a slight increase in death risk after the occurrence of adverse events was included.

Despite discussions in the literature on the methodological limitations of the evaluation of preventable adverse events based on peer review [8,10,22,23], the findings presented here emphasize not only that adverse events are prevalent but that preventable adverse events are associated with serious and irreversible harm, and even death. Notwithstanding differences in the study design, the adjusted odds ratio obtained here was much higher than that described by Garcia-Martin et al [11], who reported 1.75. According to Zegers et al [3], 10.7% of deaths among patients in the Netherlands were associated with an adverse event, while in our study this proportion was much higher (34%). In our study, cases involving preventable adverse events accounted for 26.6% of the deaths, while in the Dutch study the proportion was only 4.1% of hospital deaths [3]. These findings indicate an increased risk of serious adverse events among Brazilian patients.

### Principal study limitations

It is important to acknowledge that the findings may be related to the characteristics of the study design and the study population. Importantly, the study population was limited to admissions to 3 public teaching hospitals in a single state of Brazil, which could partially explain the results. Hospital characteristics minimize the generalization capacity of the study. Brazil has more than 5,000 hospitals; they are heterogeneous in terms of facilities, human resources, and quality of care. Since the selected hospitals have adequate medical records and develop training activities, among others factors, the results presented are probably better in these hospitals than in many others. Therefore, we consider that the numbers obtained could be underestimated for the Brazilian reality.

Primary diagnosis is an important dimension of case severity and a determinant in the process and quality of care, and thus is correlated with the occurrence of adverse events and deaths [3]. Because of the low number of cases in specific diagnostic categories, we opted to apply the Charlson index [19], without the respective weights, as indicative of the severity associated with the principal reason for admission. Despite the difficulty of interpreting the relationship between length of stay and death, case severity, and characteristics of the health service system [24], the length of stay variable, expressing severity and characteristics of the process of care, remained in the final model.

## Conclusion

In the context of health care, the evaluation of any outcome measure involves determining the extent to which the observed differences can be attributed to patient risk factors or to the various treatment processes [7]. This highlights the importance of measuring differences in the severity of cases in studies about adverse events.

Despite some limitations in the study design and analytical strategy, we observed a correlation between the occurrence of adverse events and the risk of hospital death; this association was higher when the preventability of the adverse event was considered. However, the vast majority of preventable events with fatal outcomes occurred in cases classified as level 4 in the judgment scale, and very few cases (4/25 deaths) were classified as levels with greater certainty. This point corroborates the limitations identified by Hayward & Hofer [8], i.e., that studies based on peer review are characterized by a high degree of subjectivity in the judgment of the reviewers. Even so, we feel that the current study, which includes the use of risk adjustment and multivariate models, represents an important step in the evaluation of the occurrence of adverse events. Its future developments can contribute to specific interventions and to further studies focused on improving clinical performance in hospitals [25,26], particularly considering the issue of patient safety and quality of care as part of Brazil's national healthcare agenda.

## Abbreviations

ICD-10: International Classification of Diseases, 10^th ^Revision; OR: Odds ratio; R: Pearson correlation; SPSS: Statistical Package for the Social Sciences

## Competing interests

The authors declare that they have no competing interests.

## Authors' contributions

MM contributed to the study design, statistical and data analysis, and the writing of the manuscript. CT contributed to the study design and coordination, data analysis, and the writing of the manuscript. WM contributed to the study design, interpretation, and the writing of the manuscript. ALBP participated in data analysis and the writing of the manuscript. All the authors read and approved the final manuscript.

## Pre-publication history

The pre-publication history for this paper can be accessed here:

http://www.biomedcentral.com/1472-6963/11/223/prepub

## Supplementary Material

Additional file 1 **Appendix**. Adverse event description. Click here for file
