# Supplementary material for: Hospital deaths and adverse events in Brazil
Source: BMC Health Serv Res. 2011 Sep 19;11:223. doi: 10.1186/1472-6963-11-223 (PMC3184059; doi:10.1186/1472-6963-11-223)
Supplement: Additional file 1 — Appendix. Adverse event description. [file 1472-6963-11-223-S1.RTF]

Appendix table– Adverse event description 
Case #
	Preventable adverse event	Case description	
1	Yes	71-year-old female with systemic lupus erythematosus and chronic renal failure developed sacral pressure ulcer on the 9th day of hospitalisation. 	
2	No	59-year-old hypertensive female developed sacral pressure ulcer on the 100th day of hospitalisation.	
3	Yes	87-year-old female with COPD had a ruptured gall bladder with bile leakage into the peritoneal cavity during laparoscopic colecystectomy.	
4	No	Hypertensive 66-year-old male with adenocarcinoma of the rectum underwent abdominoperineal excision of the rectum; the patient developed intestinal obstruction and was reoperated, being diagnosed with postoperative pelvic volvulus.	
5	No	Diabetic 75-year-old male underwent radical prostatectomy and developed bladder neck stenosis which required urethrotomy on the second admission.	
6	No	58-year-old female with a history of hypertension, diabetes and coronary artery disease was admitted with infected sacral pressure ulcer and lower limb ischemia resulting in dislocation of the hips. Various antibiotics were used, and after more than three weeks of hospitalisation a culture of the ulcer yielded MRSA.	
7	Yes	49-year-old female with a primary diagnosis of uterine fibroids had a wall abscess with fever, local pain and diarrhoea on the 3rd postoperative day. 	
8	No	Peritonitis following chronic peritoneal dialysis (due to lack of vascular access for haemodialysis) in a 57-year-old female patient.	
9	Yes	Hypertensive 80-year-old female underwent surgery for urinary incontinence and suffered a lesion of the uterine artery that led to interruption of the surgery. The patient was readmitted for correction of a vesicovaginal fistula.	
10	No	31-year-old female with autoimmune hepatitis underwent liver transplantation and developed a massive postoperative subcapsular liver haematoma. A revision of the anastomoses showed no problems. The patient developed major bleeding and rapidly progressive liver dysfunction leading to death.	
11	Yes	84-year-old male with metastatic adenocarcinoma of the central nervous system had pneumonia after 1 month of hospitalisation.	
12	No	36-year-old female with AIDS developed tracheal stenosis 2 months after tracheostomy performed during a previous hospitalisation.	
13	Yes	Hypertensive 87-year-old female was admitted to treat an infection of surgical wound 15 days after undergoing ostheosynthesis due to intertrochanteric femur fracture.	
14	No	42-year-old female with valvular heart disease due to rheumatic fever developed acute renal failure after 10 days using gentamicin. 	
15	Yes	Hypertensive 70-year-old male had a simple fall and developed haematomas in his arm and abdomen. The patient underwent a kidney transplant and developed infection 2 months later, with a perinephric abscess and sepsis leading to death.	
16	No	59-year-old female patient previously admitted for corneal transplant developed subconjunctival haemorrhage and was readmitted for corneal resuture one day after discharge. 	
17	Yes	45-year-old female with lumbar disc herniation developed "foot drop" following laminectomy.	
18	Yes	75-year-old male developed postoperative empyema following pulmonary segmentectomy. The patient also developed sacral and trochanteric pressure ulcers on the 21st day of hospitalisation.	
19	Yes	Diabetic 68-year-old female with dementia developed a nosocomial infection by Pseudomonas aeruginosa after 30 days of intubation due to bronchoaspiration. The patient developed a sacral pressure ulcer after 69 days of hospitalisation and immobility in bed.	
20	Yes	73-year-old male with generalized atherosclerosis had a respiratory infection on the 13th day of hospitalisation.	
21	Yes	48-year-old female had abdominal wound dehiscence following hysterectomy with adnexectomy.	
22	Yes	74-year-old male with a primary diagnosis of acute myocardial infarction had an infection of the surgical wound following coronary artery bypass grafting performed in another hospital, that progressed to sepsis and death. 	
23	No	Hypertensive 61-year-old female developed a haematoma that progressed to femoral artery pseudoaneurysm following fibrinolysis and angioplasty to treat an acute myocardial infarction.	
24	No	Generalised pruritus followed by exfoliative erythroderma in a patient that used dipyrone and paracetamol at home. 20-year-old female with a primary diagnosis of a drug hypersensitivity skin reaction.	
25	Yes	80-year female with a primary diagnosis of polypoid tumour of the duodenal papilla developed urinary tract infection after 2 weeks using a urinary catheter. 	
26	Yes	Colonic fistula on the 10th postoperative day. 69-year-old female with a primary diagnosis of tubulovillous adenoma of the splenic flexure.	
27	No	47-year-old male suffered ankle trauma with tendon injury and underwent surgical repair with a synthetic graft. Five months later the patient developed a graft infection. The graft was removed during the index hospitalisation. 	
28	Yes	Sacral pressure ulcer in a 71-year-old female with a primary diagnosis of terminal breast cancer. 	
29	No	Skin rash and facial swelling due to cyclosporine in a 45-year-old male with a primary diagnosis of Crohn's disease. 	
30	Yes	64-year-old male with a primary diagnosis of multiple trauma, including abdominal and lower extremity trauma, received first aid at another hospital and was transferred for treating an open fracture of the femur. A blood culture taken at admission showed MRSA.	
31	No	Upper extremity phlebitis following a venipuncture in a 72-year-old male with a primary diagnosis of chronic renal failure. 	
32	Yes	Uterine laceration at the surgical incision during caesarean section in a 32-year-old female.	
33	No	Wall abscess 3 days after appendectomy in a 43-year-old male.	
34	No	Retinal detachment 3 months after vitrectomy in a 75-year-old female.	
35	Yes	49-year-old male developed a haemothorax 24 hours after closed drainage of pleural empyema, progressing to shock and requiring open chest drainage and thoracostomy.	
36	Yes	Episodes of hypocalcaemia following thyroidectomy in a 58-year-old female with a primary diagnosis of thyroid nodules. 	
37	Yes	Signs of digitalis intoxication in a 63-year-old female with a primary diagnosis of chronic renal failure.	
38	Yes	Heel and sacral pressure ulcers on the 16th day of hospitalisation in an 82-year-old female with a primary diagnosis of pneumonia. 	
39	Yes	Heel and sacral pressure ulcers on the 12th day of hospitalisation in an 82-year-old male with a primary diagnosis of sepsis. 	
40	Yes	Massive subaponeurotic haematoma 5 days after a caesarean section in a 28-year-old female.	
41	Yes	Wound dehiscence following hemicolectomy, progressing with peritonitis and subphrenic abscess in a 48-year-old female with a primary diagnosis of adenocarcinoma of the colon. 	
42	Yes	Sacral pressure ulcer on the 40th day of hospitalisation in an 82-year-old male with a primary diagnosis of ischemic stroke. 	
43	Yes	Pressure ulcer on the 14th day of hospitalisation in a 40-year-old male with a primary diagnosis of infectious diarrhoea. 	
44	Yes	84-year-old male with a history of repeated syncope. The ECG showed 1st to 2nd degree atrioventricular block and left anterior fascicular block, which then progressed to complete atrioventricular block. The patient died before a pacemaker was installed. 	
45	Yes	Cellulitis at the site of vein dissection in a 69-year-old male with a primary diagnosis of lung abscess.	
46	Yes	Lymphocele 48 hours after surgery. Drainage of serosanguinous exudate, progressively more abundant on the following days. 35-year-old female with a primary diagnosis of renal transplant rejection.	
47	Yes	Clinical picture suggestive of bacteraemia in a patient with an indwelling urinary catheter. 69-year-old male with a primary diagnosis of multiple myeloma.	
48	No	57-year-old female with incisional hernia following gynaecologic surgery performed on previous hospitalisation.	
49	Yes	Headache following spinal anaesthesia for total abdominal hysterectomy in a 46-year-old female with a primary diagnosis of uterine fibroids.	
50	Yes	Sacral and heel pressure ulcers in an 80-year-old female with a primary diagnosis of liver cirrhosis. 	
51	Yes	Fall on a ramp with fracture of left radius in a 35-year-old female with a primary diagnosis of gestational hypertension.	
52	Yes	Evisceration following laparotomy for treatment of hydatidiform mole progressing to dehiscence of resuture, parietal infection and new dehiscence in a 44-year-old female.	
53	Yes	Digitalis intoxication. 63-year-old female with a primary diagnosis of pulmonary embolism.	
54	No	Puncture of the carotid artery during an attempt to puncture the jugular vein. 63-year-old male with a primary diagnosis of cirrhosis from hepatitis C.	
55	Yes	Aponeurosis dehiscence following appendectomy in a 68-year-old male.	
56	No	53-year-old female with a primary diagnosis of uterine fibroids experienced abdominal pain following hysterectomy. Ultrasound imaging showed a blood collection in the abdominal wall. 	
57	No	Haematoma at episiotomy site in a 43-year-old female.	
58	No	Retrosternal haematoma following heart surgery. 42-year-old male with a primary diagnosis of combined aortic and mitral lesions.	
59	Yes	Upper gastrointestinal bleeding with a drop in haematocrit (42 to 15 in 5 days); the patient received no blood replacement. 78-year-old female with a primary diagnosis of Fournier's syndrome.	
60	No	Diarrhoea as a possible consequence of the use of a broad-spectrum antibiotic (third-generation cephalosporin). 68-year-old female with a primary diagnosis of Kartagener syndrome.	
61	Yes	75-year-old male with a diagnosis of Hodgkin's lymphoma 4 months earlier. The patient was admitted for chemotherapy and died 15 days after admission with no chemotherapy and no record of cancer staging.	
62	Yes	Intra-abdominal abscess following caesarean section in a 38-year-old female.	
63	No	Sacral pressure ulcer in a 67-year-old female with a primary diagnosis of renal abscess. 	
64	Yes	Untreated urinary infection detected during prenatal care. Urinalysis showed pH 6, ketonuria 3+, haemoglobinuria 3+, 6 to 8 cells, 15 to 20 leukocytes/field and increased bacterial flora in a 25-year-old female. No urine culture was ordered or suggested, and the patient received no therapeutic guidance during prenatal care.	
65	Yes	Headache, vomiting and neck stiffness. No complete clinical and neurological examination or analysis of CSF performed during the entire hospitalisation, resulting in missed diagnosis of possible cerebral toxoplasmosis in a 37-year-old male with a primary diagnosis of AIDS..	
66	Yes	Prolongation of hospital stay due to the appearance of foul-smelling secretion at the surgical wound 7 days after surgery. Culture yielded P. mirabilis and Enterobacter cloaceae. Treatment was started with piperacillin and tazobactam for two days and then changed to ciprofloxacin.	
67	Yes	74-year-old female with a primary diagnosis of severe coronary artery disease and chronic renal failure had a haematocrit of 28.8% at admission. At the end of the hospitalisation (24 hours before death) the haematocrit was 22%. No red blood cell concentrate was prescribed during hospitalisation. 	
68	Yes	Face injury resulting from simple fall due to syncope secondary to severe anaemia. 35-year-old female with a primary diagnosis of uterine fibroids.	
69	No	Significant haematoma at the site of episiotomy with spontaneous resolution in a 37-year-old female.	
70	Yes	Wound dehiscence at intestinal loop progressing with sepsis, following surgical removal of pancreatic cyst in a 55-year-old male.	
71	Yes	Swelling and redness at the surgical wound 3 days after caesarean section in a 33-year-old female.	
72	Yes	Abdominal aortic aneurysm demonstrated by abdominal CT, not treated surgically during the 39 days of hospitalisation, in a 78-year-old male.	
73	Yes	Urinalysis showing leukocytosis – 20 leukocytes/field on the 20th day of hospitalisation. 71-year-old female with a primary diagnosis of chronic renal failure.	
74	Yes	63-year-old female with a primary diagnosis of cervical tumour was admitted with significant anaemia and vaginal bleeding. Suspected uterine tumour (an ultrasound performed 2 months earlier showed small fibroids). A biopsy showed cervical tumour. The patient continued to bleed and to receive blood replacement. 	
75	No	Surgical site infection following laryngectomy. 69-year-old male with a primary diagnosis of cancer of the larynx.	
76	Yes	Sacral pressure ulcer in an 80-year-old female with a primary diagnosis of hydronephrosis. 	
77	No	Wound dehiscence with resuture 11 hours later, 1 day after cataract surgery. 68-year-old female with a primary diagnosis of senile cataract.	
78	Yes	Nosocomial lung infection 1 month after admission. X-ray showed consolidation in the middle third of the lung. 72-year-old male with a primary diagnosis of abdominal mass of unknown origin.	
79	Yes	Stenosis of jugular vein catheter requiring puncture of the subclavian vein. 53-year-old female with a primary diagnosis of decompensated lower extremity ischemia.	
80	Yes	Sacral and heel pressure ulcers on the 3rd week of hospitalisation. 63-year-old male with a primary diagnosis of liver cancer with lung metastases.	
81	Yes	67-year-old male with a primary diagnosis of acute renal failure admitted to the ER with urinary bladder distension..No record of diagnostic investigation; the patient was discharged without a plan for continuity of care.	
82	No	Wound dehiscence at the lower third of the surgical wound. 41-year-old male with perforated stenotic rectal tumour, purulent peritonitis and multiple liver metastases.	
83	Yes	Acute renal failure caused by the use of a nephrotoxic antibiotic (gentamicin), which was maintained at full dose. 83-year-old male with a primary diagnosis of cholangitis.	
84	No	Haematuria following placement of a urinary catheter. 74-year-old male with a primary diagnosis of ischemic stroke.	
